# From Nutcracker Phenomenon to Nutcracker Syndrome: A Pictorial Review

**DOI:** 10.3390/diagnostics11010101

**Published:** 2021-01-11

**Authors:** Antonio Granata, Giulio Distefano, Alessio Sturiale, Michele Figuera, Pietro Valerio Foti, Stefano Palmucci, Antonio Basile

**Affiliations:** 1 Nephrology and Dialysis Unit, “Cannizzaro” Hospital, 95026 Catania, Italy; antonio.granata4@tin.it (A.G.); asturiale@gmail.com (A.S.); 2 Radiology Unit I, Department of Medical Surgical Sciences and Advanced Technologies “GF Ingrassia”, University Hospital “Policlinico—San Marco”, University of Catania, 95123 Catania, Italy; pietrofoti@hotmail.com (P.V.F.); spalmucci@sirm.org (S.P.); basile.antonello73@gmail.com (A.B.); 3 Radiology Unit II, University Hospital “Policlinico—San Marco”, 95123 Catania, Italy; micfiguera@tiscali.it

**Keywords:** nutcracker syndrome, renal, nutcracker phenomenon, renovascular hypertension, CT, ECD

## Abstract

Left renal vein (LRV) entrapment, also known as nutcracker phenomenon if it is asymptomatic, is characterized by abnormality of outflow from the LRV into the inferior vena cava (IVC) due to extrinsic LRV compression, often accompanied by demonstrable lateral (hilar) dilatation and medial (mesoaortic) stenosis. Nutcracker syndrome, on the other hand, includes a well-defined set of symptoms, and the severity of these clinical manifestations is related to the severity of anatomic and hemodynamic findings. With the aim of providing practical guidance for nephrologists and radiologists, we performed a review of the literature through the PubMed database, and we commented on the definition, the main clinical features, and imaging pattern of this syndrome; we also researched the main therapeutic approaches validated in the literature. Finally, from the electronic database of our institute, we have selected some characteristic cases and we have commented on the imaging pattern of this disease.

## 1. Background

Any vessel can be compressed by adjacent anatomic structures (bone, organs, and other vessels), or they may cause compression of adjacent hollow viscera. One of the possible combinations of anatomical abnormalities and abdominal vessel entrapment syndromes can result in left renal vein (LRV) entrapment [1,2]. Referring to entrapment of the LRV, the terms nutcracker syndrome (NS) and nutcracker phenomenon (NP) have often been used interchangeably in older literature [3], but since LRV entrapment does not always take on clinical significance, currently the term nutcracker syndrome seems more appropriate to refer to conditions with clinical expression [4]. The aim of this work is to provide a critical review of the acquisitions in the international literature relating to LRV compression syndrome, defining relevant imaging patterns for diagnostic purposes, providing the clinician with an address on when and how to use the terms nutcracker syndrome (NS) or nutcracker phenomenon (NP), and what to expect from imaging. For this purpose, in October 2020, we performed a search through the PubMed databases in the fields of abdominal vessel imaging and abdominal vascular entrapment syndromes. We used the following keywords: “nutcracker syndrome”, “nutcracker phenomenon”, “LRV entrapment”, and “LRV compression”, and we examined titles and abstracts. We included in our analysis reference guidelines, previous revisions, meta-analyses, and case reports with features of exceptional rarity for which it has been possible to access the entire content. We excluded irrelevant articles (case reports or series with non-exceptional characteristics), recurrent articles from the same authors, and articles written in other languages. Based on these inclusion and exclusion criteria, we selected a total of 53 articles that we consulted for the preparation of this paper, all listed in our reference list. In addition, we provide a pictorial review of the most important radiological patterns in order to provide a diagnostic address for radiologists and nephrologists.

## 2. Definition

Left renal vein (LRV) entrapment is an anatomical condition characterized by extrinsic compression of the renal vein and consequent altered outflow in inferior vena cava (IVC), with demonstrable dilation of the hilar region and narrowing of the para-aortic region of the renal vein. The term nutcracker phenomenon is used to refer to this specific anatomical condition when it is not associated with symptoms. Nutcracker syndrome, on the other hand, is the term used to define the LRV entrapment associated with a well-defined set of symptoms, and the severity of these clinical manifestations is related to the severity of anatomic and hemodynamic findings [4,5,6]. There is no unanimous agreement on what symptoms are severe enough to warrant the designation of a clinical syndrome. NS can be caused by compression of the LRV between the aorta and the superior mesenteric artery (SMA)—in this case it will be appropriate to speak of anterior nutcracker syndrome—or by the rarer compression of the LRV between vertebral bodies and the aorta—posterior nutcracker or pseudo-nutcracker syndrome [7].

## 3. Epidemiology

The first anatomic description of LRV entrapment was reported in 1937 by Grant [8], while the first clinical impact of this anatomic condition was made by El-Sadr and Mina in 1950 [9]; as far as we know, the term nutcracker was probably first used in Schepper’s papers in 1972 [10] and Chait’s [11] in the following year [3]. Entrapment of the LRV does not seem to be a genetically determined phenomenon, and family cases are exceptionally reported in the literature [12]. The exact incidence of this anatomic variant is not defined [13], and it is considered underdiagnosed, as it is often pauci-symptomatic or completely asymptomatic. The symptomatic forms that fall under the definition of nutcracker syndrome are reported in the literature with a bimodal peak, with most cases identified in adolescence or young adulthood (second or third decade), and then again in the third to fourth decades of life [14], with higher prevalence in the female sex [15]; a low body mass index has been shown to correlate positively with NS, because thin subjects are predominantly exposed to alteration of the aortomesenteric angle [16].

## 4. Pathogenesis and Mechanism of LRV Entrapment

The LRV is between 6 and 10 cm long, and receives blood from the ipsilateral adrenal gland, gonadal vein(s), and lumbar veins. Along its course (in most cases), the LRV passes inferiorly to the SMA and anterior to the aorta. Anatomic variants of this vascular configuration must be taken into account, since these vessels have valves that prevent blood reflux, allowing the flow only toward the inferior vena cava. Incompetent valves or absent or ectopic venous outlets may increase the pressure within the LRV favoring the reflux. In addition to the anatomic variants, the individual’s somatic characteristics must also be taken into account. Low body mass index is regarded as a risk factor for LRV entrapment [16] because of two different proposed mechanisms both related to paucity of retroperitoneal adipose tissue: it can reduce the meso-aortic angle and it can cause dorsal migration of the kidney and renal pelvis (i.e., posterior renal ptosis) [3].

NP may also arise due to anatomical variations, such as an LRV that is more cephalic upon union with the inferior vena cava and, as such, is immediately inferior to the SMA or an SMA that instantly descends, determining a very short angle with abdominal aorta.

In most individuals, the aorto-mesenteric angle (AMA) is between 38° and 65°, while an AMA < 35° is compatible with the anatomical condition underlying the entrapment of the LRV, and may be associated with abnormal outflow from the LRV into the IVC, with a significant increase in the pressure gradient between the LRV and IVC [13]. Other causes of LRV compression include pancreatic neoplasms, para-aortic lymphadenopathy, retroperitoneal tumors, aortic aneurysms, or fibro lymphatic tissue between the SMA and aorta [16], but the terms NP and NS should not be used in connection with the compression of the LRV from these causes [1].

**Figure 1:**
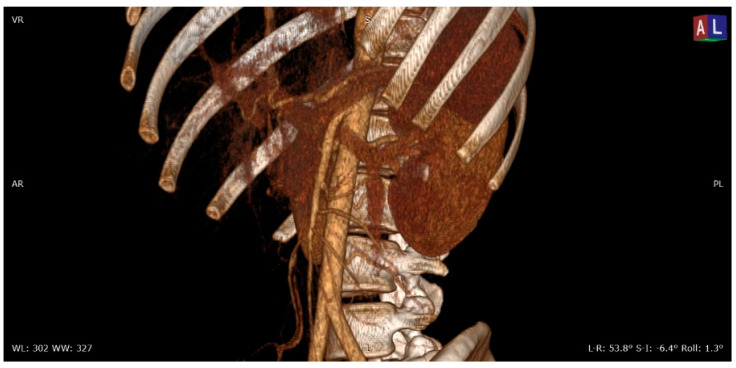
3D volume rendering (VR) contrast enhanced CT scan (portal venous phase) shows the relationships between the aorta, the superior mesenteric artery, and the left renal vein in a patient with nutcracker syndrome; in this acquisition, an initial enhancement of the left gonadal vein is also appreciable, which appears dilated (varicocele).

**Figure 2:**
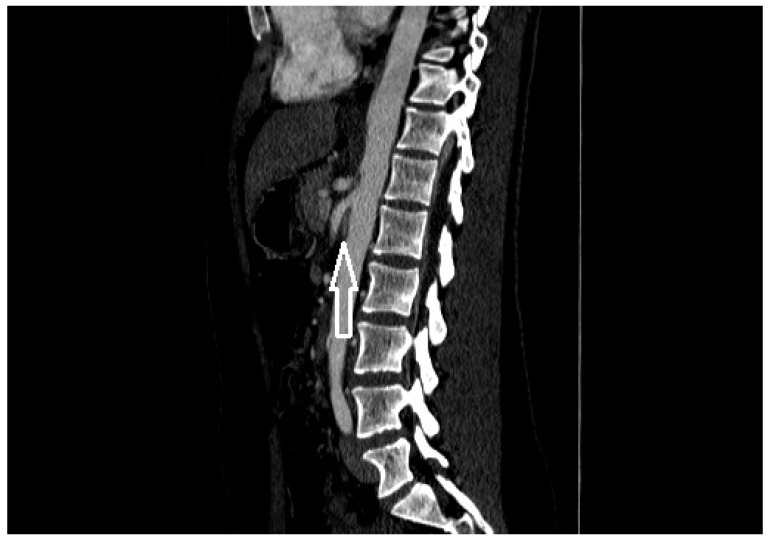
A patient with nutcracker syndrome: contrast enhanced CT scan during arterial phase, with sagittal reconstruction; in this image, it is possible to appreciate the entrapment of the left renal vein (white arrow) between the superior mesenteric artery and the aorta.

On the basis of anatomic presentation, the NP is classified into two types: anterior and posterior. Anterior NP is described as a result from compression of the LRV between the SMA and abdominal aorta [17] (Figure 1 and Figure 2), while posterior NP refers to left renal venous hypertension secondary to compression of the retro aortic LRV between the abdominal aorta and the vertebral column [18]. Other rarer forms of entrapment of LRV are possible: Basile et al. described a case of compression of the LRV by an aberrantly ventral right renal artery at the renocaval junction with dilated lumbar varices [19]; Polguj et al. described anatomic variations of the anterior NP in which the compressive component is determined by a right renal artery emerging abnormally [17]. Combined (anterior and posterior) NS was also reported in a patient with duplication of the LRV [20]. Both anatomic configurations can determine a common clinical picture characterized by varicose veins of the renal pelvis and left gonadal vein dilatation. This reflects clinical symptoms quite comparable to flank pain, hematuria, and varicocele or abnormal menstrual bleeding [21].

## 5. Clinical Features Suspicious for NS

It has to be kept in mind that NP is an anatomical condition that is detected with diagnostic imaging investigations, while NS is a clinical diagnosis characterized by symptoms in the presence of LRV entrapment. In the literature, there are several clinical pictures ascribable to entrapment of the LRV, and it is believed that the symptoms are related to the increase in pressure in the LRV [1]; however, there is no consensus on how severe these symptoms should be to indicate that the pathology is NS [1]. 

**Figure 3:**
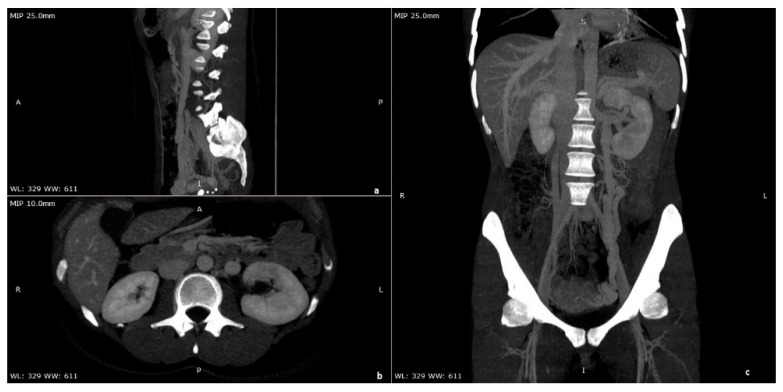
Maximum intensity projection (MIP) = 25 mm and multi-planar reformation (MPR), (**a**) sagittal, (**b**) axial, and (**c**) coronal contrast enhanced CT portal venous phase images of a young woman affected by nutcracker syndrome with pelvic congestion syndrome, acquired in portal venous phase; in (**a**) and in (**c**), pathological serpiginous dilatation of the left ovarian vein is shown.

**Figure 4:**
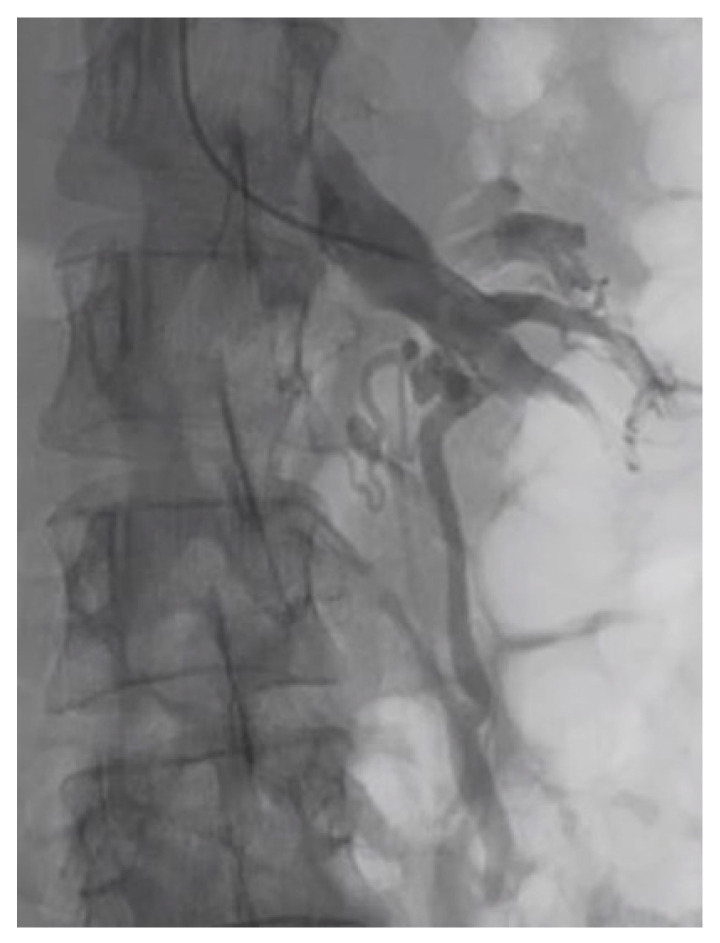
Another patient with nutcracker syndrome and pelvic congestion syndrome; angiographic acquisition shows left pelvic varicocele, characterized by appreciable dilatation of the left ovarian vein.

Hematuria is the most frequent sign in NS patients. The pathogenetic mechanism proposed to explain hematuria is that increased venous pressure into the LRV and left gonadal vein can lead to rupture of the septa between the venules and the collecting system in the renal parenchyma. On the other hand, no specific glomerular damage is reported in the literature [13,22]. Increased pressure determines back congestion of the left gonadal vein that is at the base of men’s left varicocele [23]. In women, pelvic congestion syndrome, which may include dysmenorrhea, dysuria, dyspareunia, and pelvic pain, has been reported [24] (Figure 3 and Figure 4). In some cases, vulvar, gluteal, and lower extremity varices have been noted [25]. It is unclear if there is a correlation between renin dependent hypertension and NS. A case of renin-dependent hypertension has been reported that reversed after endovascular vein stent placement [26], but an original work that examined the excretion of renin and aldosterone taken from the left and right renal vein in patients affected by NS didn’t find any significant differences in the secretion of renin and aldosterone between the two renal veins [27]. In the absence of a different indication from the literature, we can assume that there is no relationship between hypertension and NS.

## 6. Diagnostic Criteria for NP

**Figure 5:**
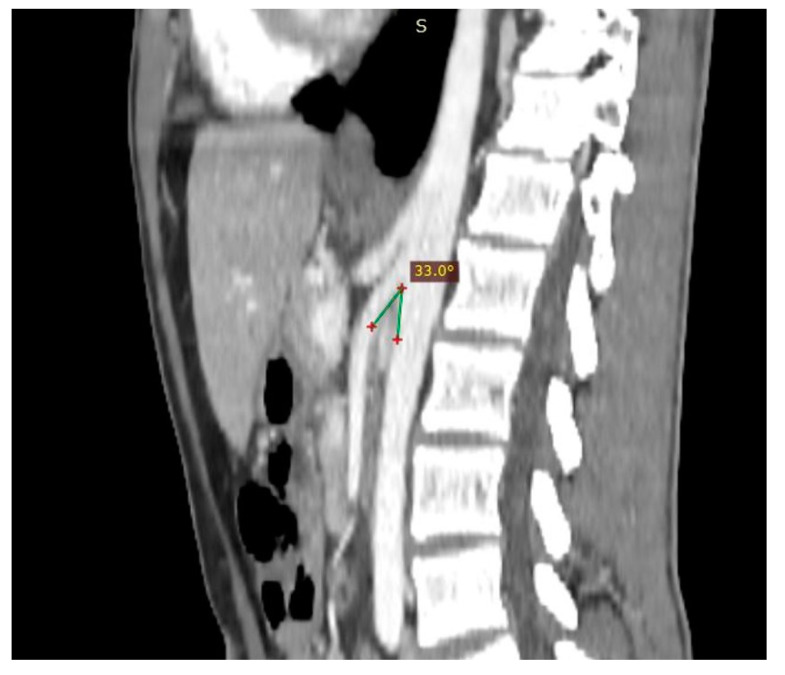
Patient affected by nutcracker syndrome. The sagittal reconstruction demonstrates an aorto-mesenteric angle of about 33°, lower than the norm (aorto-mesenteric angle normally ranges from 38° to 56°).

**Figure 6:**
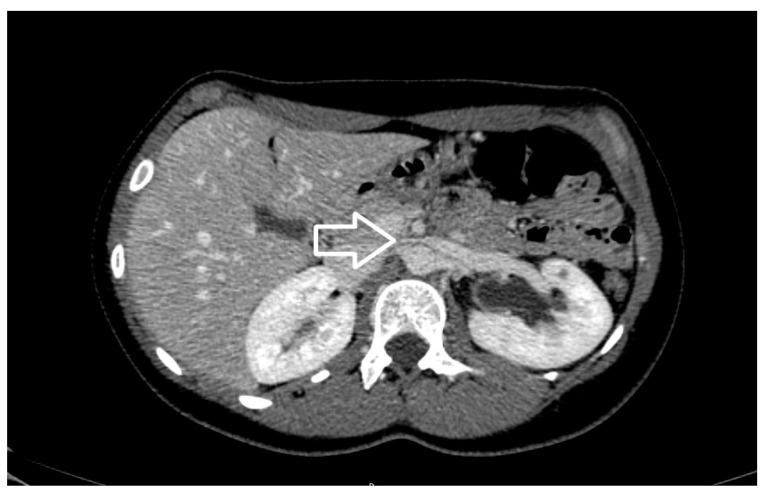
Axial view of contrast enhancement CT scan demonstrating the beak sign (white arrow), caused by the compression of the left renal vein from the aorta and superior mesenteric artery.

**Figure 7:**
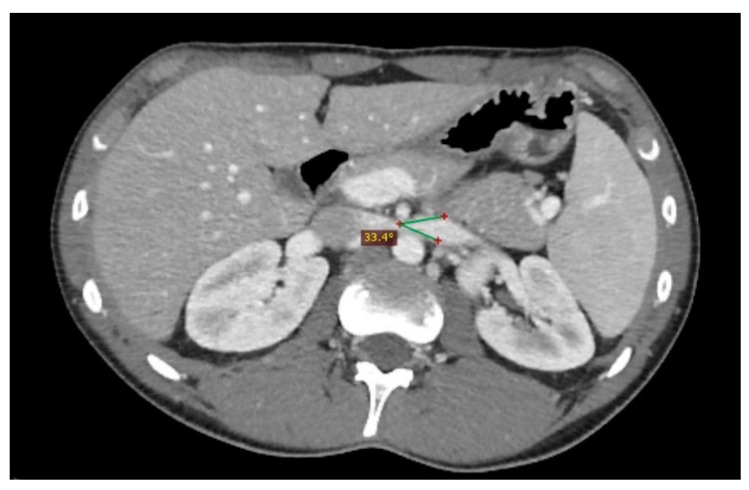
Another nutcracker syndrome patient; in this axial image, it is possible to appreciate a beck angle higher than 32°, suggestive of entrapment of the left renal vein.

**Figure 8:**
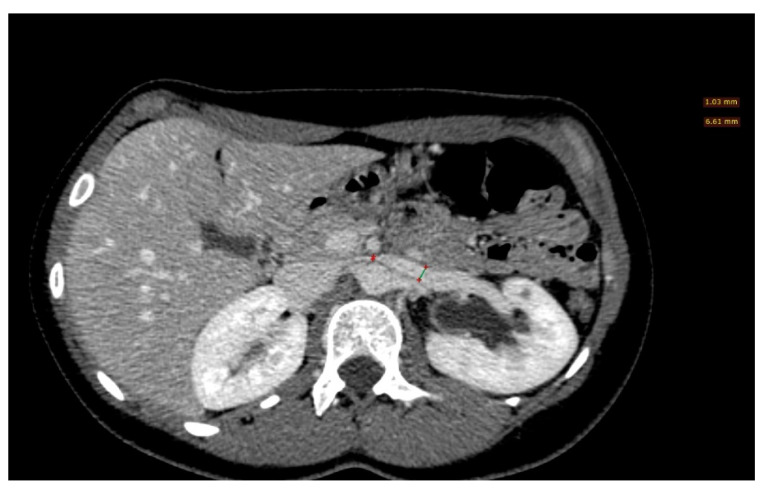
A left renal vein diameter ratio higher than 4.9 is specific for nutcracker syndrome; it is necessary to measure the axial diameter of the renal vein at the renal hilum and in correspondence with the stenosis between the superior mesenteric artery and the aorta.

A contrast-enhanced computed tomography (CT) with both arterial and portal venous phase or a magnetic resonance imaging (MRI) using angiography sequences can be used to study the anatomy of the LRV and the relationships with the surrounding structures [1,2]. Both tests allow highlighting of the mesenteric artery origin from abdominal aorta and the compression and stenosis of the LRV; coronal and sagittal reconstructions also allow the characterization of the left gonadal vein and collateral circulation with lumbar veins. With sagittal reconstruction, it is possible to evaluate the AMA, which is the angle between the aorta and the SMA; AMA normally range from 38° to 65° [1,28], and is on average lower in patients affected by anterior NS, up to 14.5° [29]. An AMA lower than 35° is compatible with the diagnosis of entrapment of the LRV and, therefore, with NP or NS (Figure 5) [28]. In axial CT images, the most important signs are: the beck sign, which is the stenosis of the LRV between the aorta and the SMA (Figure 6), the beck angle, which is significant for the diagnosis of NP if it is <32° (Figure 7), and finally an LRV diameter ratio (hilar to aorto-mesenteric ratio) more than 4.9, which has a positive predictive value of 100% (Figure 8) [3].

**Figure 9:**
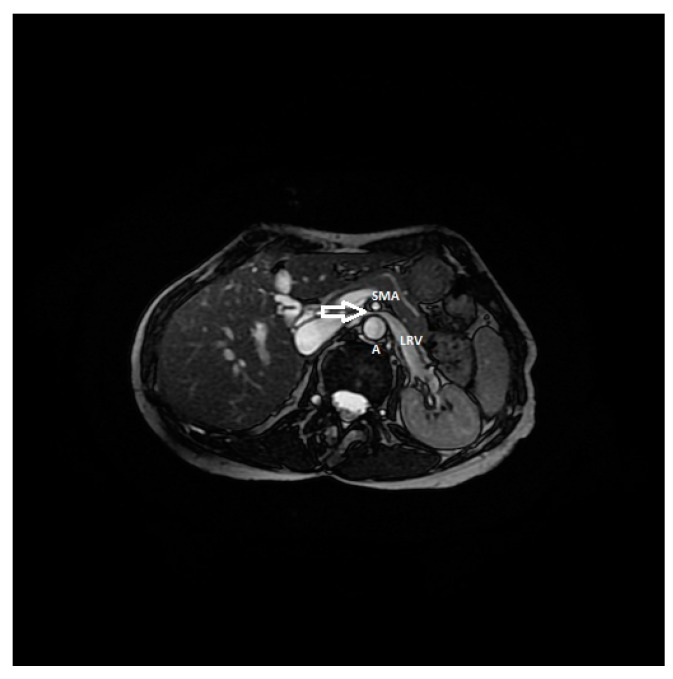
RMI, axial FIESTA sequence, shows the compression of the left renal vein between the aorta and superior mesenteric artery in a patient affected by nutcracker syndrome with flank pain and pelvic congestion syndrome. In this image, the beck sign (white arrow) is highlighted; SMA: superior mesenteric artery, A: aorta, LRV: left renal vein.

**Figure 10:**
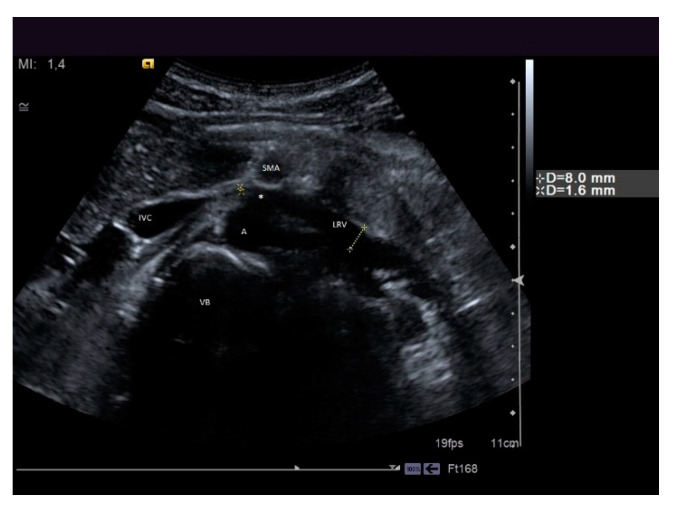
A patient suffering from flank pain, varicocele, and hematuria evaluated at the ultrasound (US) laboratory of the Nephrology and Dialysis Unit. The axial grayscale sonogram demonstrates the anatomical relationships between the left renal vein, aorta, and superior mesenteric artery, with stenosis at the aorto-mesenteric angle (beck sign) and dilation of the hilar section of the left renal vein with a ratio of nutcracker phenomenon. A: aorta, SMA: superior mesenteric artery, LRV: left renal vein, IVC: inferior vena cava, VB: vertebral body, white asterisk: beck sign.

**Figure 11:**
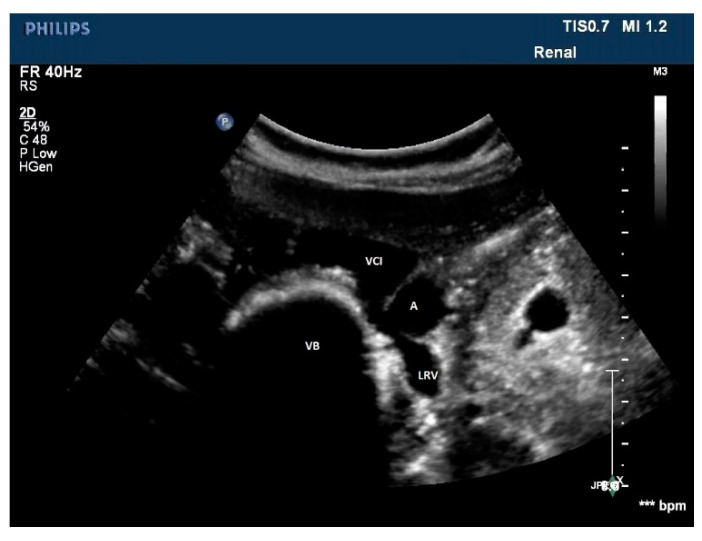
A rare case of posterior nutcracker syndrome evaluated with US; in this axial image, it is possible to recognize the left renal vein that runs between the abdominal aorta and the vertebral column. A: aorta, LRV: left renal vein, IVC: inferior vena cava, VB: vertebral body.

Important findings demonstrated by MRI closely resemble findings using 3D-CT with similar levels of accuracy (Figure 9), and include dorsolateral torsion of the left kidney, abnormally high course and compression or pre-stenotic dilatation of the LRV, abnormal configuration of SMA, and peri-renal or gonadal vein varices [25,30]. For the posterior NS, obviously, there is not a defined angle criterion [31]. Even an ultrasound examination (US) performed by an expert operator can demonstrate the same specific findings for NP on axial and coronal planes (for example, the beck sign, LRV dilatation, and characteristic LRV diameter radio), and allows the addition of functional characteristics through the eco-color Doppler (ECD-US) evaluation (Figure 10 and Figure 11). Zhang et al. described two ECD criteria for the diagnosis of NS: (1) the flow velocity of stenosis of the LRV in the supine position accelerates remarkably, and the acceleration, which is more than 100 cm/s, is more obvious after the patient has stood for 15 min; and (2) the inner diameter ratio between the renal hilum and stenosis of the LRV in the supine position is >3, and is >5 after the patient has stood for 15 min [30]. As reported by Cheon et al., a peak systolic velocity ratio greater than 4.7 has a sensitivity of 100% and a specificity of 90% for diagnosis [32]. It is important to note that US may be the first diagnostic imaging test in outpatients with nephrological symptoms, and may address subsequent investigations. Angiography represents an invasive investigation useful for demonstrating morphological (such as LRV stenosis and dilated collateral vessels) and functional alterations. Retrograde venography is used to measure the reno-caval pressure gradient, and, through the injection of contrast medium, to characterize the dilation of the left gonadal vein and any side of peri-ureteral and pelvic venous circles [4]. The LRV to IVC pressure gradient is less than 1 mm Hg in healthy subjects, and (although a wide range of gradient values has been published) an elevated gradient > 3 mm Hg between the LRV and the IVC can be used as a diagnostic criterion for NCP [22,32,33,34,35,36,37].

According to Hohenfellner, NP is defined when the size of the LRV is reduced by more than 50% as it passes through the aorto-mesenteric clamp [36]. The measurement is made on a longitudinal section of the vein and by measuring the maximum and minimum range. It shall be considered a gauge of 4–5 mm [36]. The reno-caval pull back gradient is helpful in establishing the diagnosis. Some analytical diagnostic parameters have been summarized in Table 1.

## 7. Treatment

In NS patients, treatment is variable, and conservative management, interventional endovascular approaches, or more invasive surgical treatments are possible; the choice of whether to proceed with invasive treatment or not depends on the severity of the symptoms and clinical signs [38]. Conservative treatment is recommended for patients with modest hematuria and who are under 18 years of age, and any procedure should follow at least six months of conservative follow up [13]; it has been reported that in most patients with mild symptoms, there may be complete spontaneous resolution of symptoms [39]. There is no consensus on recommended pharmacological therapy for patients with NS; in mild cases, no therapy seems to be needed. It appears that renal perfusion could benefit from the administration of aspirin and that the administration of angiotensin-converting enzyme inhibitors (RAS-I) can help to relieve orthostatic proteinuria; alacepril has been recommended as the best compared with analogous RAS-I [13,39,40].

When clinical symptoms of NS are not tolerable and result in significant clinical manifestations (chronic pain, recurrent hematuria, pelvic congestion), conservative treatment may not be enough. Many surgical approaches, including medial nephropexy, renal vein bypass, transposition of the left renal vein, transposition of the superior mesenteric artery, gonad-caval bypass, and auto-transplantation of the left kidney, were reported [41,42]. Ullery et al. reported a small experience of transposition of the LRV in pediatric patients with good short-term results [43]. Recently, Yun et al. proposed a new robotic-assisted surgical procedure for transposing the LRV, with encouraging results on a small sample of patients [7]. Despite the surgical approach, hematuria can be persistent, and it may still be necessary to perform a nephrectomy. In this case, renal auto-transplantation, which involves a wider surgical exposure, an additional arterial anastomosis, and longer renal ischemia, is much more invasive than LRV transposition [43].

**Figure 12:**
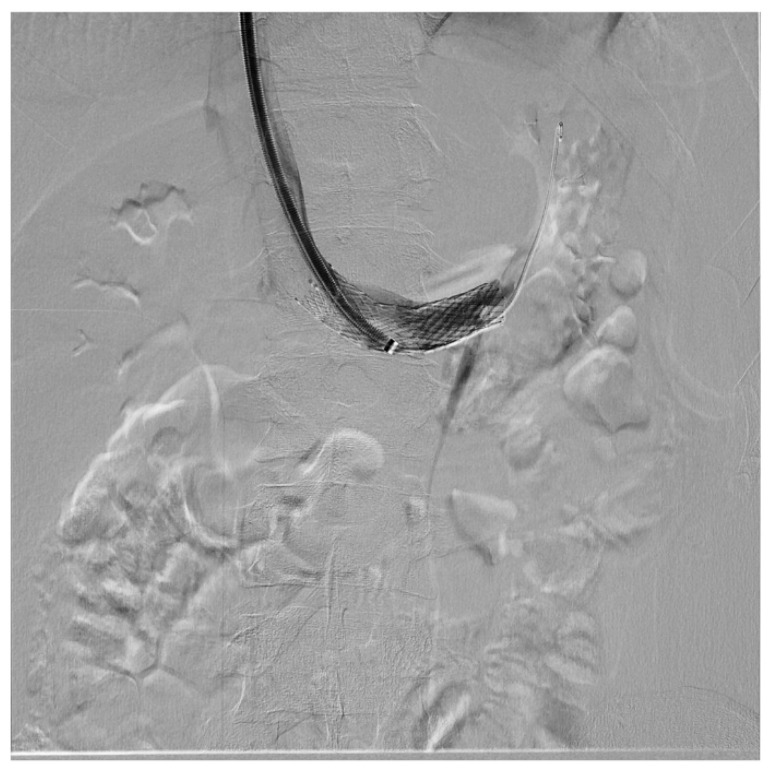
Patient affected by nutcracker syndrome (with nutcracker phenomenon, flank pain, hematuria, and left varicocele) treated with endovascular procedures; diagnostic phlebography documented a significant stenosis of the left renal vein at the aorto-mesenteric clap; at the stenosis, a stent (visible in the image) dilated with a balloon catheter was released.

**Figure 13:**
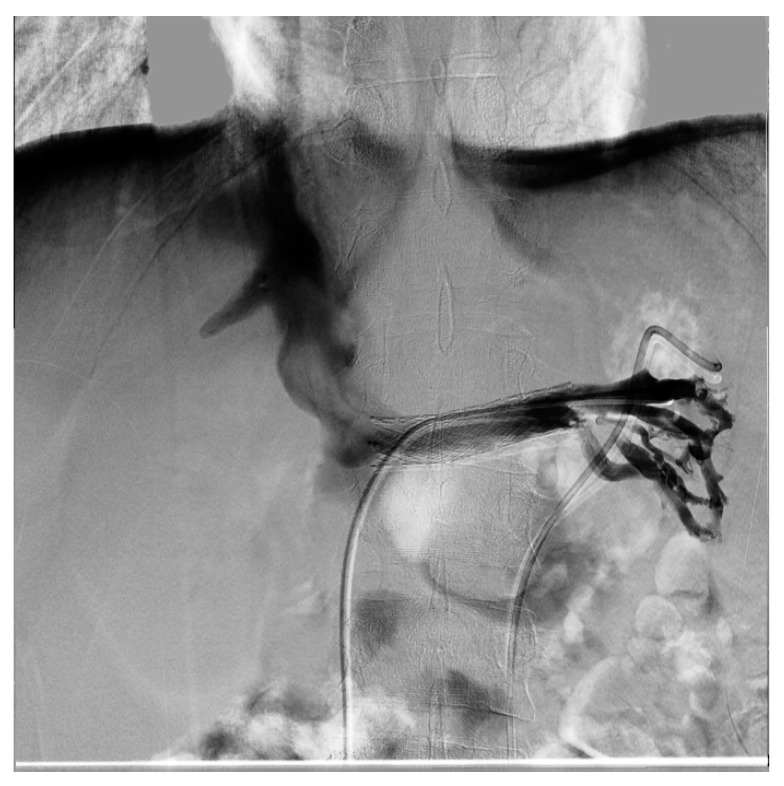
Same patient as the previous figure, re-evaluated a year later for a new episode of hematuria; diagnostic phlebography documents that the stent is in the normal position and is normally patent; collateral circulation or varicose ectasias are not recognizable.

**Figure 14:**
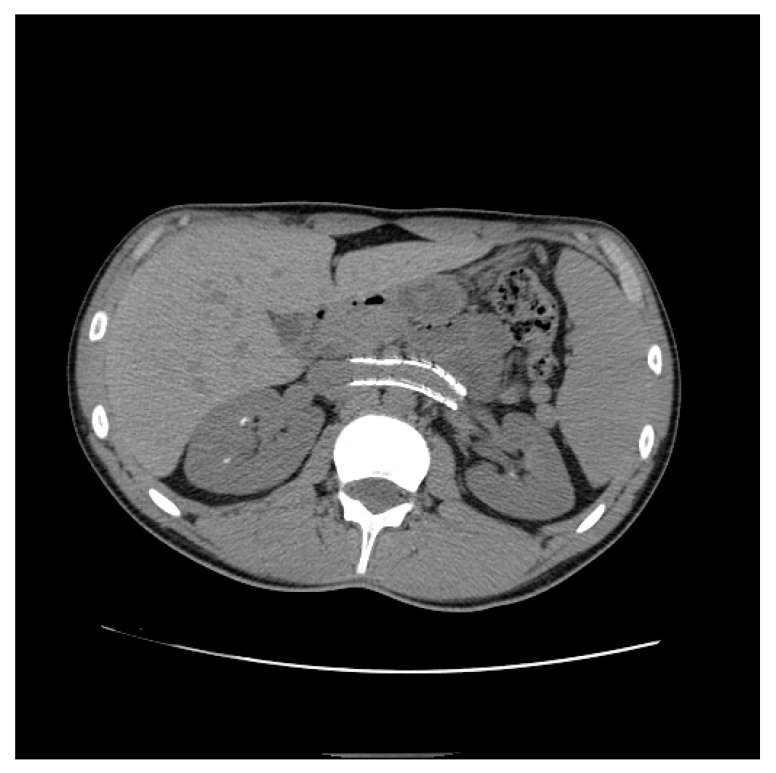
Patient who previously underwent endovascular treatment with symptom resolution, subjected to imaging investigations for other reasons. Non-contrast axial CT passing through aorto-mesenteric clamp demonstrated the correct positioning of a stent in the left renal vein with resolution of the stenosis.

Endovascular treatment has now assumed a prominent role. After the first report in 1996 by Neste et al. [44], endovascular surgery becomes more important, because it is a minimally invasive procedure. In a series by Wang et al., 30 patients were treated with a self-expanding nitinol stent and (in three cases) whit gonadal vein embolization; authors declared that technical success was achieved in all of the patients, with improvement of symptoms and without any post-operative complications [45]. Hartung et al. reported a small series of five female patients who were treated for NS, and described that there were two cases of stent dislodgement with secondary recurrence of the symptoms, suggesting use of long stents protruding into the inferior vena cava to prevent this secondary failure of treatment [46]. Basile et al. reported a series of three young patients treated with a nitinol self-expandable stent, with complete resolution of symptoms and a normal CD-US pattern and without complications after a prolonged follow-up (18 months) [47] (Figure 12, Figure 13 and Figure 14).

In a case of stenosis of the LRV caused by an aberrant right renal artery, double stenting solved retrograde filling of lumbar collaterals [48]. As far as we know, there are no shared guidelines on the most correct methodology for positioning the stents and choosing the right device, but the opinions of experts are reported in the literature based on the available evidence. Policha et al., in order to minimize the risk of stent migration, recommend the use of a stent oversizing of 20% compared to the diameter of the vessel to engage the stent at the level of the first order branch of the renal vein, and the use of balloon-expandable stents over self-expanding stents, while no role is recognized in primary venoplasty [49]; the same authors attempted the use of intravascular ultrasound (IVUS) to obtain precise measurements of the LRV, without success [49].

Many patients refuse an invasive surgical treatment, opting for endovascular procedures. However, these procedures are not free from complications. The most important (for incidence and potentially damage) is stent migration, while in-stent restenosis and venous occlusion resulting from fibromuscular hyperplasia or thrombosis rarely occur. Anticoagulant and antiplatelet treatment is necessary to reduce the risk of thrombosis [50].

Some authors have proposed the ligation of the left gonadal vein laparoscopically to reduce pelvic congestion syndrome caused by NS [51]. It is not clear if this procedure worsens hematuria and the other renal symptoms. 

## 8. Conclusions

LRV entrapment is an anatomical condition that can run completely asymptomatic (in this case it is appropriate to define it only as NP) or be responsible for a set of symptoms that determine the so-called NS. Both the clinician and radiologist treating a patient with flank pain or intermittent hematuria should also consider LRV entrapment syndrome in the differential diagnosis, especially when these symptoms are not related to a specific kidney disease. Diagnosis requires imaging evaluation of the first (CD-US) and second level (CT or MRI). Several surgical approaches have been proposed, and it is currently believed that endovascular procedures, which are even more tolerated by patients, are associated with lower intraoperative risk and less post-operative complications, with more rapid remission.

## Figures and Tables

**Table 1:** Diagnostic criteria for nutcracker phenomenon; details are given in the text. AMA: aorto-mesenteric angle, CT: computed tomography, ECD-US: eco-color Doppler, IVC: inferior vena cava, LRV: left renal vein, MRI: magnetic resonance imaging, NP: nutcracker phenomenon, PVR: peak velocity ratio, US: ultrasound.

Criterion	Instrumental Examination Technique	Reference Values Compatible with the Diagnosis of NP	References
AMA	CT (post contrast arterial and portal venous phase, sagittal reconstruction), MRI (angiography sequences, sagittal acquisition), US (b-mode, sagittal scans)	<35°	[28]
Beck angle	CT (post contrast arterial and portal venous phase, axial scans), MRI (angiography sequences, axial acquisition), US (b-mode, axial scans)	<32°	[3]
LRV diameter ratio		>4.9	[3]
PVR	ECD-US (Doppler sampling in correspondence with the hilar region and stenotic region of the LRV)	>4.7	[32]
LRV to IVC pressure gradient	Invasive sampling of venous pressure values in correspondence with the LRV (distal to the stenosis) and in the IVC	>3 mmHg	[22,32,33,34,35,36,37]

## Data Availability

The data presented in this study are available on request from the corresponding author.

## Abbreviations

AMA	aortomesenteric angle
CIV	common iliac vein
ECD-US	eco-color Doppler
IVC	inferior vena cava
IVUS	intravascular ultrasound
LRV	left renal vein
MALS	median arcuate ligament syndrome
MRI	magnetic resonance imaging
NS	nutcracker syndrome
NP	nutcracker phenomenon
OVS	ovarian vein syndrome
PV	peak velocities
RAS-I	angiotensin-converting enzyme inhibitors
SMA	superior mesenteric artery
TC	computed tomography
UPJ	uretero-pelvic junction
US	ultrasound
